# Innovate or die!: Genomic data and the electronic health record (EHR)

**DOI:** 10.1016/j.atg.2014.09.007

**Published:** 2014-10-02

**Authors:** Rebecca Fein

**Affiliations:** Laboratory Informatics Institute, 2400 Lake Park Drive, Suite 435, Smyrna, GA 30080, USA

## Abstract

The sharing of patient data, such as test results, is important for appropriately treating patients. Inclusion of this data allows for clinical error prevention, particularly if a new clinician becomes involved. If genetic information, including family history or genetic test results, is not accessible to treating physicians, care may suffer. Innovation is needed to address the problem of lack of access to important data, such as genomic test results, not structured to fit into an EHR.

Without data integration care will, and does suffer. Innovations, albeit long in the making (Blatt, 2013), are improving the care of patients. The next step forward is better utilization of available information, including genomic test results.

## 1 Introduction

Electronic health records (EHR) are a commonly used vehicle to share patient information.

In the absence of an EHR, a patient with increased risk for heart disease in her 20s, as determined by family history and a genetic test result that is not documented in the EHR, may not be given appropriate care by her primary care physician (PCP) if he/she is lacking access to the genomic test results. Integrating genomic results into the EHR is clearly necessary to incorporating the benefits of genomic knowledge to provide better care, enable prevention, provide better/more targeted care, and ultimately improve outcomes.

Some challenges to integrating genomic data into the EHR (Williams, 2012):

• Inadequate education of clinical workforce as it relates to genomic information.
• Insufficient evidence to demonstrate benefit from this integration.
• Concerns about privacy, and security of the data.
• Concerns about provider payment.

Medical schools do not teach doctors how to interpret genetic/genomic information. Instead physicians receive narrative reports containing result interpretations, often with indications for clinical action, which importantly are not structured for integration in the patient's EHR Scanning in a report does not enable other physicians easy access to potentially important information for care decisions. The lack of easy access to genetic/genomic information is, and will increasingly be, an important care issue.

## 2 The need to innovate

Innovation is typically met with fear and skepticism, especially in an industry such as healthcare.

Disruptive innovation is required to create the kind of shake up needed for resistance to fade and change to take hold. A revolutionary process that allows for seamless integration of genomic results and family history, into an EHR and for that information to be used by clinicians to inform their decision making processes.

## 3 What types of innovation can help?

Innovation is the only way to solve the challenges related to integration of genomic data into the EHR.

Innovations that enable storing and sharing test results in EHRs are necessary to ensuring that genomic medicine will have the desired outcomes. It is important that innovative solutions take into account the needs of patients, clinicians, and others who may be involved in the care process (such as insurance companies). One possible solution could be a physician alert when an increased risk of an adverse condition, such as the increased risk of heart disease mentioned in the beginning of the commentary, is identified through genomic test result and family history. In order to be of use for clinical decision-making, genomic information must be clear, complete, relevant, timely, and actionable. Vendors would also need to be involved in this process. They need to create products that allow for the improved storage and management of data, instead of side by side, or narrative reporting, they could create an EHR that allowed for a more expansive report/interpretation if needed, but could allow the clinician and patient to clearly see the connection between the data, such as test results from genetic testing and the patient's overall healthcare needs.

Innovations that train and connect medical students with genomic information and interpretation of that information could be valuable for ensuring that newly minted physicians are knowledgeable about the types of information obtained by different types of tests. Given that studies will continue to update information available and interpretations of data (such as test results), this is a good skill for genomic medicine physicians to have.

A great example of how genomic data can be used to accomplish these goals within an EHR is currently underway at Vanderbilt University Medical Center (DeGaspari, 2014). Vanderbilt has developed a pharmacogenomic resource for enhanced decision in care and treatment (PREDICT) project (DeGaspari, 2014). PREDICT was implemented in 2010 as part of Vanderbilt's quality improvement initiative and since then has genotyped more than 14,000 patients (DeGaspari, 2014). This project uses advanced EHR and clinical decision support (CDS) tools, to assist clinicians with genome-informed decision making (DeGaspari, 2014).

This program has allowed 370 clinicians to find patients that are at risk for side effects or other undesirable outcomes from seven types of drugs, with sufficient data on prescribing and genetic risk factors identified (DeGaspari, 2014). These clinicians found that 88.3% of patients tested had some form of genetic risk related to these drugs (DeGaspari, 2014). These are patients that can now have a better standard of care, because their clinicians are away of the risk related to their body's processing of a particular drug commonly used to treat their condition. Clinicians at Vanderbilt, when they find a risk, have processes in place for selecting an alternative drug therapy (DeGaspari, 2014). This is an example of how innovations related to genomic data can assist patients and clinicians in improving patient outcomes, and also in cutting costs of care through the avoidance of unnecessary (and possibly harmful) usage of drugs (DeGaspari, 2014).

PREDICT could serve as a template for other innovations, because it only uses drugs that have enough information for genomic risk to be seen, and it is allowing clinicians across a network to improve the outcomes of patients, while avoiding costs related to adverse reactions. This type of innovation could be beneficial to the healthcare system as a whole, but especially to the rural and critical access patients, who may need to drive for miles to pick up prescriptions and see doctors for care related to adverse events.

## 4 Conclusion

The demand for genomic medicine is not going to go away, genomic data is here and it is here to stay (Blatt, 2013). Innovative solutions to enable genomic data to reside in EHRs are urgently needed, less we create a bottleneck of millions of terabytes of clinically relevant data that has no way of entering the EHR. It is important that the industry innovates to provide better service to patients, and give clinicians the tools they need for using all clinically relevant information (such as test results).

The road to innovation in healthcare has many challenges, some of which were outlined in this commentary. The alternative to innovation is to be left behind in a graveyard of obsolescence. The consequences for patients are far worse, for patients the burden of not creating innovative solutions is poor quality patient care, which in some cases will result in death due to some genetic/genomic data that is not presented in an actionable manner in the EHR.

## References

[bb0005] Blatt, M., World Wide Medical Director, Intel, personal, communication, 6/11/13.

[bb0010] DeGaspari J. (2014). The 2014 Healthcare Informatics Innovator Awards: Third Place Winner: Vanderbilt University Medical Center. The 2014 Healthcare Informatics Innovator Awards: Third Place Winner: Vanderbilt University Medical Center.

[bb0030] Williams M.S. (2012). The public health genomics translation gap: what we don't have and why it matters. Public Health Genomics.

